# Crosstalk between alternative splicing and inflammatory bowel disease: Basic mechanisms, biotechnological progresses and future perspectives

**DOI:** 10.1002/ctm2.1479

**Published:** 2023-11-20

**Authors:** Chentao Zou, Xinquan Zan, Zhenyu Jia, Lu Zheng, Yijie Gu, Fei Liu, Ye Han, Chunfang Xu, Airong Wu, Qiaoming Zhi

**Affiliations:** ^1^ Department of Gastroenterology The First Affiliated Hospital of Soochow University Suzhou China; ^2^ Department of General Surgery The First Affiliated Hospital of Soochow University Suzhou China

**Keywords:** alternative splicing, basic mechanisms, biotechnological progresses, inflammatory bowel disease, perspectives

## Abstract

**Background:**

Alternative splicing (AS) is an omnipresent regulatory mechanism of gene expression that enables the generation of diverse splice isoforms from a single gene. Recently, AS events have gained considerable momentum in the pathogenesis of inflammatory bowel disease (IBD).

**Methods:**

Our review has summarized the complex process of RNA splicing, and firstly highlighted the potential involved molecules that target aberrant splicing events in IBD. The quantitative transcriptome analyses such as microarrays, next‐generation sequencing (NGS) for AS events in IBD have been also discussed.

**Results:**

Available evidence suggests that some abnormal splicing RNAs can lead to multiple intestinal disorders during the onset of IBD as well as the progression to colitis‐associated cancer (CAC), including gut microbiota perturbations, intestinal barrier dysfunctions, innate/adaptive immune dysregulations, pro‐fibrosis activation and some other risk factors. Moreover, current data show that the advanced technologies, including microarrays and NGS, have been pioneeringly employed to screen the AS candidates and elucidate the potential regulatory mechanisms of IBD. Besides, other biotechnological progresses such as the applications of third‐generation sequencing (TGS), single‐cell RNA sequencing (scRNA‐seq) and spatial transcriptomics (ST), will be desired with great expectations.

**Conclusions:**

To our knowledge, the current review is the first one to evaluate the potential regulatory mechanisms of AS events in IBD. The expanding list of aberrantly spliced genes in IBD along with the developed technologies provide us new clues to how IBD develops, and how these important AS events can be explored for future treatment.

## Graphical abstract

Our review have summarised aberrant splicing events and mechanisms in the pathogenesis of IBD.

The progresses of AS detection in IBD patients are systematically described, and challenges of technological innovation are also raised.

These AS‐related genes provide us novel clues how IBD develops, and how these important AS events can be further elucidated for future treatment.

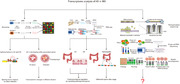

## 1 INTRODUCTION

Inflammatory bowel disease (IBD) is a chronic inflammatory‐mediated disorder, which is clinically characterised by ulcerative colitis (UC), Crohn's disease (CD) and some other conditions.^1^ In clinical practice, the main features of gastrointestinal tract inflammation in IBD patients include episodes of diarrhoea, bloody stools, abdominal pain, weight loss, fever and other immune‐related extraintestinal manifestations.^1^, ^2^, ^3^ As the disease progresses, most of IBD patients may experience some more severe complications, including strictures, abscesses, fistulas, perforation and others, which may cause significant morbidity. Notably, long‐term colitis in patients with IBD can also lead to colon cancer called colitis‐associated cancer (CAC).^4^, ^5^ IBD is now considered as an unignorable life‐threatening disease in people of all ages, including children, adults, pregnant and geriatric populations, and often significantly reduces the quality of life. More depressingly, the global morbidity of IBD has increased rapidly over the past 20 years from developing and recently developed countries.^6^, ^7^, ^8^, ^9^, ^10^ Over the past decades, considerable progresses have been made to greatly improve our knowledge of the pathophysiology of this disease. It is thought that IBD results from the individual's genetic susceptibility, defects in barrier function and an aberrant and continuing immune response to intestinal microbial flora.^1^, ^11^ Besides, other factors, including autophagy, reactive oxygen species production, endoplasmic reticulum stress and metabolic pathways associating with cellular homeostasis are also closely correlated with the development of IBD.^12^, ^13^ Recent studies using multi‐sampling and data integration combined with other novel techniques, such as whole‐genome sequencing, RNA sequencing, single nucleotide polymorphism arrays, genome‐wide methylation analysis and meta‐analyse, can help us better explain the cause of IBD.^14^, ^15^, ^16^, ^17^ However, we still have not fully elucidated this complex pathogenetic process. Therefore, it is an urgent need for investigators to reveal the detail pathogenesis of IBD and prevent the development of this costly and disturbing disease.

**FIGURE 1:**
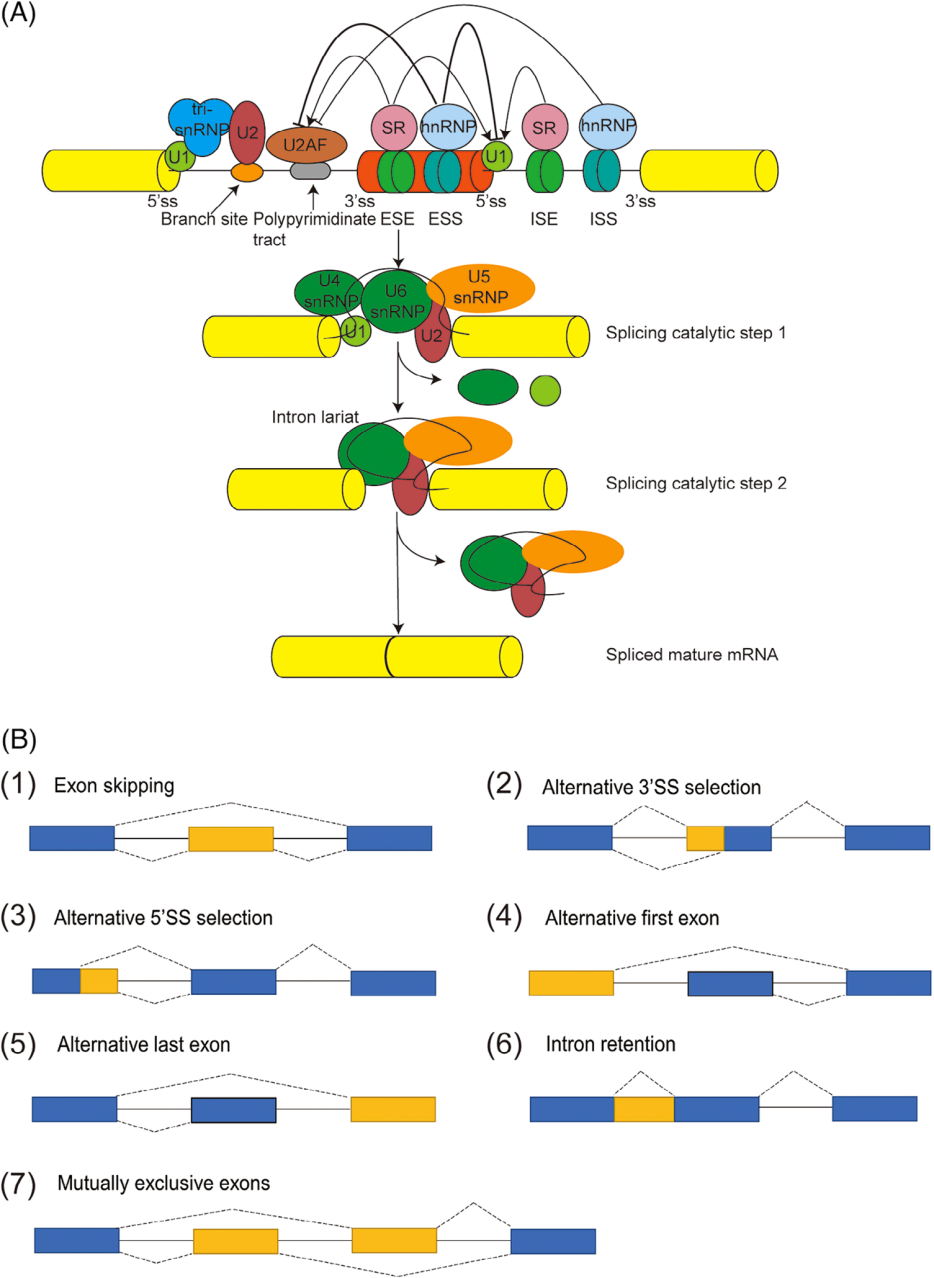
The process and diverse patterns of AS. (A) The splicing process undergoes sequential phosphodiester transfer reactions, which is catalysed by spliceosomes, including five snRNPs U1, U2, U4, U5 and U6 as well as splicing factors. The snRNAs cooperate to recognise the splice sites and catalyse the two steps of splicing reaction. (B) The diverse patterns of AS include exon skipping, alternative 3′SS selection, alternative 5′SS selection, alternative first exon, alternative last exon, intron retention and mutually exclusive exons.

Alternative splicing (AS) is an omnipresent regulatory mechanism of gene expression that enables the generation of diverse splice isoforms from a single gene,^18^ which contributes to the diversity of proteomes in >90% of human genes. During the constitutive splicing, each intron will be removed and the remained exons can be joined together to produce a mature mRNA. Compared with the constitutive splice, AS is far more complex. The process is performed by the spliceosome, which is a big complex consisting of 5 ribonucleoproteins (RNPs) involving the small nuclear RNA U1, U2, U4, U5, U6 and multiple auxiliary proteins cooperating to precisely recognise the splicing sites and catalyse the two splicing reaction steps.^18^, ^19^ First of all, the splicing process starts with the identification of the 5′ splicing site by the snRNP U1 and the combination of the splicing factor 1 (SF1) with the branch point 3 and of the U2 auxiliary factor (U2AF) heterodimer with the 3′ terminal AG and polypyrimidine tract. This assembly contributes to the E complex formation, which can be transformed to an ATP‐reliant, pre‐spliceosome A complex after replacing SF1 with the U2 snRNP at the branch site. Subsequently, the recruitment of U4/U6–U5 tri‐snRNP complex causes the B complex formation. These changes of complex contain the release of U1 and U4 and the formation of intron lariat which is known as the result of the first splicing catalytic step. Finally, the excised intron lariat is degraded, and U2, U5 and U6 snRNPs can be released to produce mature mRNA, which is known as the result of the second splicing catalytic step.^19^, ^20^ In addition, the splicing of pre‐mRNA is also regulated by splicing regulatory factors that serve as regulatory proteins and bind to the position which are able to enhance or silence the splicing process. There are four binding domains termed exonic splicing silencers, intronic splicing silencers, exonic splicing enhancers and intronic splicing enhancers.^21^ Two primary families of splicing regulatory factors are characterised by heterogeneous nuclear RNPs (hnRNPs) or Ser/Arg‐rich proteins (SRs), which exert either inhibitory or activating effects on the recognition and usage of binding site (Figure 1A).^22^, ^23^ As shown in Figure 1B, the main AS patterns are divided into seven types: exon skipping (also called cassette exon), alternative 3′splice site selection, alternative 5′splice site selection, alternative first exon, alternative last exon, intron retention and mutually exclusive exons.^18^, ^21^

In normal circumstances, AS can be heavily regulated. However, like most pathophysiological processes, AS is open to faults, which can change the functions of proteins and lead to a vast repertoire of diseases. For instance, AS has been proved to account for multiple neurodegenerative disorders, such as Alzheimer's disease, Parkinson's disease and spinal muscular atrophy.^24^, ^25^ ‘Angiogenesis’ is defined as the constitution of new capillary blood vessels from pre‐existing micro vasculatures and regulated by a variety of factors.^26^, ^27^ In 2019, Bowler et al.^28^ uncovered the potential AS mechanisms in angiogenesis and summarised the alternative spliced isoforms of essential genes that were involved in the process of angiogenesis. Defects in AS are frequently found and closely associated with the occurrence of human tumours, and abnormal changes of AS can also affect the cancer progression.^29^, ^30^ Recently, a study from Zhou et al.^31^ preliminarily discussed all splicing defects both in adults and paediatric IBD. Our review have summarised the complex process of RNA splicing, and first highlighted involved molecules that target aberrant splicing events from a view of the basic mechanisms of IBD. The expanding list of aberrantly spliced genes in IBD along with the developed technologies provide us novel clues to how IBD develops, and how these important AS events can be further elucidated for future treatment.

## 2 HOW AS EVENTS PARTICIPATE IN THE PATHOGENESIS OF IBD?

### 2.1 AS and intestinal microbiota

The mammalian gastrointestinal tract can be considered as a suitable habitat for an enormous and interconnected community of microorganisms. The complex aggregation of microbes in gut, including fungi, viruses, protozoans and bacteria, is termed as intestinal microbiota. A great deal of clinical and experimental data have confirmed that alterations in microbial communities can promote the intestinal damage and play a primary role in the occurrence, progression and treatment of IBD.^32^, ^33^ The gut microbiota composition in IBD patients has been reported to be remarkably distinct from that of healthy individuals.^34^, ^35^ Moreover, the gut microbiota‐originated metabolites, including bile acids, short‐chain fatty acids, tryptophan metabolites and signals from microbial metabolites, can help opportunistic pathogens colonise and invade to the gut, increase the risk of dysregulated host responses and finally lead to the initiation of IBD.^36^, ^37^, ^38^

**FIGURE 2:**
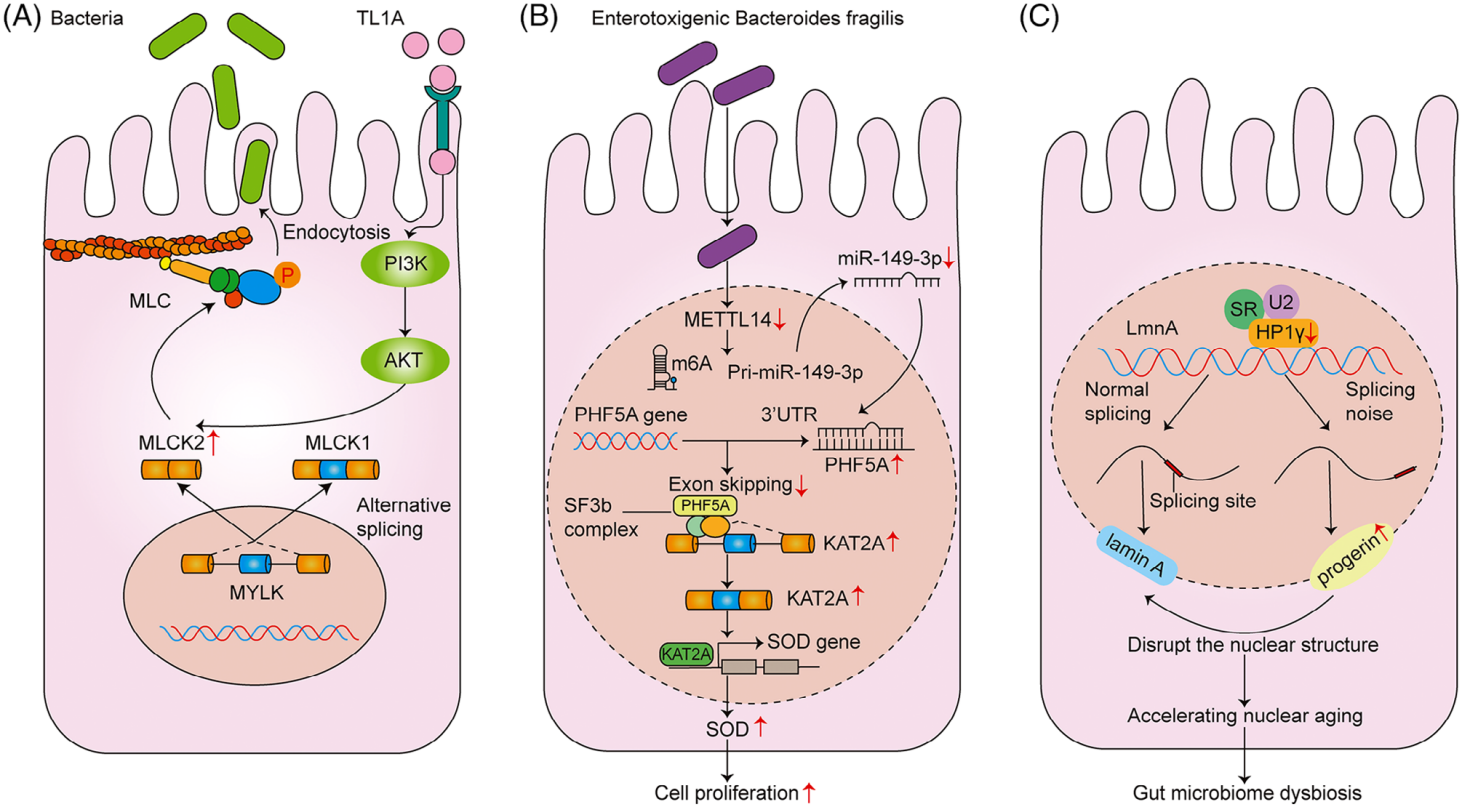
AS and intestinal microbiota. (A) The pro‐inflammatory cytokine TL1A activates the PI3/AKT pathway and up‐regulates the MLCK splice variant (MLCK2), which finally leads to MLCK‐mediated terminal web contraction, and invokes bacterial internalisation. (B) ETBF‐infected epithelial cells can down‐regulate miR‐149‐3p depending on the METTL14‐mediated N6‐methyladenosine methylation, increase the PHF5A expression and promote the RNA alternative splicing of KAT2A. The exon 8 of KAT2A is less frequently skipped after ETBF‐treatment, and can directly binds to the SOD2 promoter region, thus transactivating SOD2 and leading to cell proliferation. (C) The HP1γ gene inactivation is proved to broadly increase splicing noise, lead to more opportunities of lamin A mRNA splice variants (progerin), and finally result in gut homeostasis rupture and trigger the IBD‐like traits.

Notably, several studies have found that defects in RNA splicing are also linked to the dysbiosis of intestinal microbiota and contribute partly to the pathogenesis of IBD. Intestinal epithelia express two myosin light chain kinase (MLCK) splice variants (the full‐length and shorter isoform MLCK2). In IBD patients, the pro‐inflammatory cytokine tumour necrosis factor (TNF)‐like 1A (TL1A) was demonstrated to activate the phosphatidylinositol 3‐kinase/protein kinase B (AKT) signals and up‐regulate the MLCK splice variant (MLCK2), which might induce the MLCK‐mediated terminal web contraction, and invoke bacterial internalisation (Figure 2A).^39^ Cao et al.^40^ showed that *Enterotoxigenic Bacteroides fragilis* (ETBF, a subtype of *B fragilis*) was closely relevant to the occurrence of IBD and CAC. ETBF‐infected cells could significantly down‐regulate miR‐149‐3p depending on the METTL14‐mediated N6‐methyladenosine methylation, which further increased the PHD finger protein 5A (PHF5A, a splicing modulator interacting with SF3b complex) expression, and promoted the RNA AS of Lysine acetyltransferase 2A (KAT2A) in CRC cells. The exon 8 of KAT2A was less frequently skipped after ETBF‐treatment and directly bound to the superoxide dismutase 2 (SOD2) promoter region, thus transactivating SOD2 and leading to cell proliferation (Figure 2B).^40^ Heterochromatin Protein 1γ (HP1γ) is a protein which can safeguard the RNA splicing accuracy in the intestinal epithelium and reduce the impact of naturally occurring non‐canonical spicing events (spicing noise). In UC patients and mice, the HP1γ gene inactivation was proved to broadly increase splicing noise, lead to more opportunities of lamin A mRNA splice variants (progerin) and finally result in gut homeostasis rupture and trigger the IBD‐like traits (Figure 2C).^41^

### 2.2 AS and intestinal epithelial barrier function

The gastrointestinal mucosa constitutes an essential barrier that serves as nutrient and fluid absorption as well as secretion. Defending the barrier integrity plays an essential role in the regulation of immune system. The healthy mucosal barrier comprises the mucus layer, epithelial cells and junctional complexes.^42^ The mucus layer is considered as the first‐line physical defence that consists of highly glycosylated mucin proteins and limits exposure to all threats, including intestinal chemical and biological pathogens, to epithelial cells. Intestinal epithelial cells (IECs) are the central barrier, which can form a physiochemical protection that separates the microbes and antigens from the host's internal milieu. These IECs are derived from a pool of pluripotent stem cells at the bottom of the crypts and ultimately differentiated into goblet cells, Paneth cells, microfold cells, absorptive enterocytes and enteroendocrine cells. Together with the mucus layer and cellular immune system, IECs are crucial and associate with each other via a range of intercellular junctions, including adherens junctions (AJs), tight junctions and desmosomes.^43^, ^44^ Over the past decade, there have been increasing recognitions that either defects or breakdowns of the intestinal epithelial barrier function have been observed in many intestinal disorders such as IBD.^45^, ^46^

**FIGURE 3:**
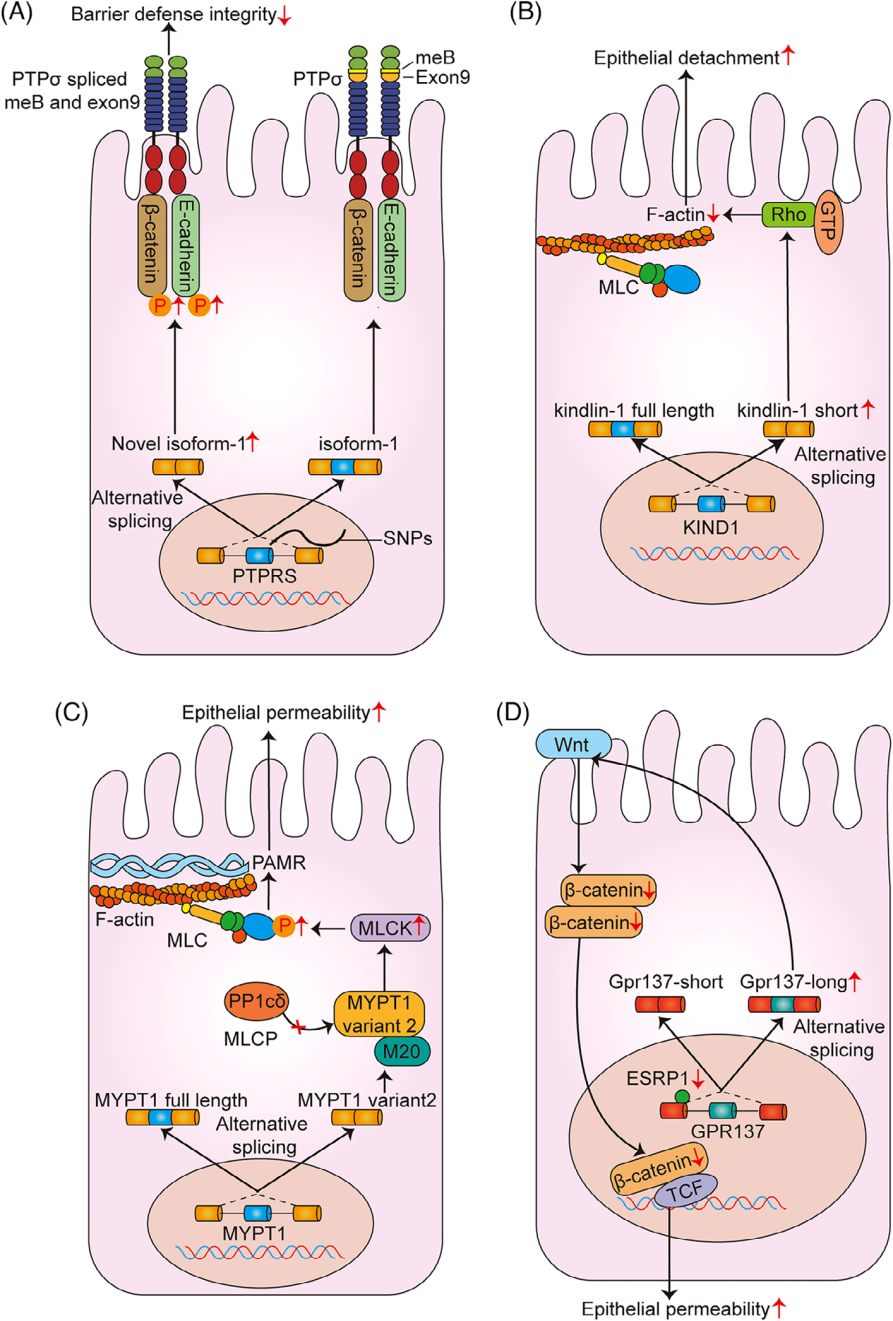
AS and intestinal epithelial barrier function. (A) Three SNPs (rs17130, rs886936 and rs8100586) that flanked exon 8 in the PTPRS gene induce alternative splicing of exon 9 and meB. Remove of the third immunoglobulin like domain of PTPσ alters the ligand binding or recognition for E‐cadherin and β‐catenin, which lead to the decomposition of adherens junction. (B) The Kindlin‐1 short isoform impairs the interactions with RhoA, and causes the epithelial detachment and defect of intestinal barrier. (C) The variant 2 of MYPT1 lacks the binding affinity to the catalytic substrate of MLCP, increase the MLCK activity and MLC phosphorylation, and subsequently triggers the PAMR contraction‐mediated switch of intestinal epithelial permeability. (D) An elevated level of the long version of GPR137 differently mediates the Wnt/β‐catenin signalling pathway, thus impairing intestinal barrier integrity and increasing the susceptibility to colitis or CAC.

Of note, a weakened intestinal epithelial barrier caused by abnormal AS events is also closely associated with the susceptibility to IBD. As early as 2007, receptor protein‐tyrosine phosphatase sigma (PTPRS) has been proved as a susceptibility gene for IBD by the animal and genetic studies. E‐cadherin and β‐catenin are two essential AJ proteins that maintain the barrier defence in the gut and also serve as the colonic substrates for protein tyrosine phosphatases (PTPs). In human IBD, three SNPs (rs17130, rs886936 and rs8100586) that flanked exon 8 in the PTPRS gene were found to result in potential alternate splicing of exon 9 and meB, which could entirely remove the third immunoglobulin like domain of PTPσ and alter the ligand binding or recognition for E‐cadherin and β‐catenin. Subsequently, the E‐cadherin and β‐catenin phosphorylation caused the redistribution of E‐cadherin and cell disassembly, which finally led to the decomposition of AJ (Figure 3A).^47^ Kindlin‐1 is a focal adhesion protein that contributes to the activation of integrin receptors. Previous data have found that there are two Kindlin‐1 transcripts (5.8 and 4.9 kb) in murine and human colon, which can correspondingly encode the 43 kDa kindlin‐1 and full‐length 74 kDa protein isoform, respectively. In 2007, Kern et al.^48^ reported that the Kindlin‐1 short isoform might impair the interactions with ras homolog gene family member A (RhoA), cause the epithelial disconnection and defect of intestinal barrier and seem to be an event sequence in the pathogenesis of Kindler syndrome‐linked colitis (Figure 3B). Besides, the regulation of intestinal epithelial permeability and barrier loss is identified to require the MLCK and myosin regulatory light chain (MLC) phosphorylation.^49^ Myosin phosphatase target subunit 1 (MYPT1) is a housekeeping gene, and IECs can express both isoforms (full length and variant 2 of MYPT1). Compared with the full‐length isoform, the variant 2 of MYPT1 could reduce the binding affinity for the myosin light chain phosphatase (MLCP). Thus, the dominant variant 2 might increase the MLCK activity and MLC phosphorylation and subsequently trigger the perijunctional actomyosin ring (PAMR) contraction‐mediated switch of intestinal epithelial permeability in IBD (Figure 3C).^50^ In 2017, Mager et al.^51^ also discussed that the novel importance of epithelial splicing regulator protein 1 (ESRP1, an AS regulator) in the colitis and altered colorectal cancer development. In humans, ESRP1 expression was significantly reduced in the biopsies taken from patients with IBD, and the low level of ESRP1 was closely related with a poorer outcome for CRC patients. Mechanistically, G protein‐coupled receptor 137 (GPR137) was identified as a newly splicing target of ESRP1, and a low level of ESRP1 as well as an elevated level of the long version of GPR137 could differently mediate the Wnt/β‐catenin signalling pathway, thus impairing intestinal barrier integrity and increasing the susceptibility to colitis or CAC (Figure 3D).^51^

### 2.3 AS and innate immune

The pathogenesis of IBD remains elusive, but IBD appears to be related to underlying excessive immune responses against the microorganisms of the intestinal flora, including the innate and adaptive immunity.^52^ The innate immune response is non‐specific, quick and represents the first line of defence against pathogens within a short time. In the case of innate immune response cells, a large variety of different types, including neutrophils, macrophages, monocytes, myeloid‐derived suppressor cells, innate lymphoid cells, IECs, dendritic cells and natural killer cells, are critical and play a pivotal role in the active phase of IBD.^53^

**FIGURE 4:**
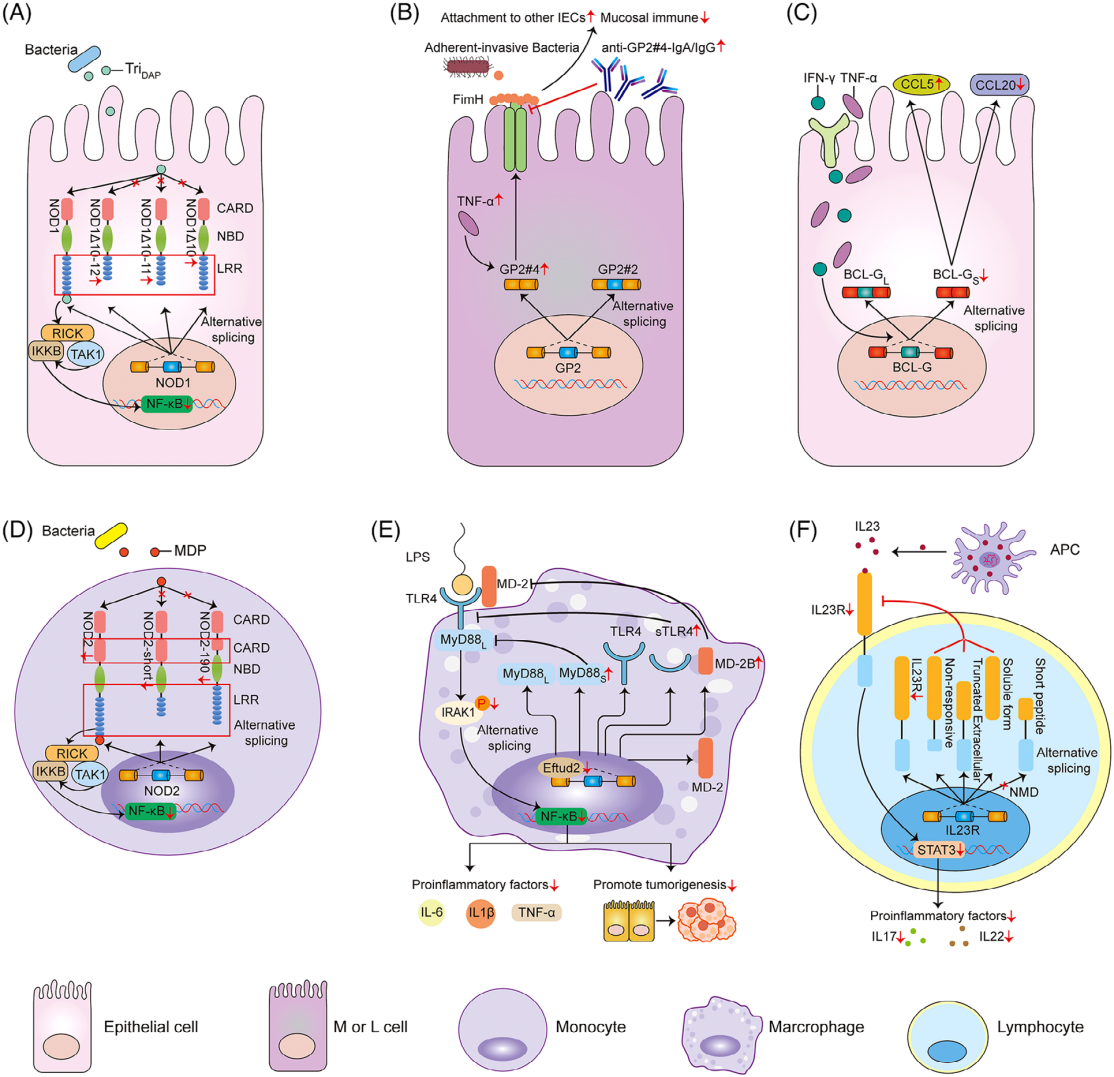
AS and innate immune. (A) In IBD, only other three splicing variants but not the full‐length of NOD1 are up‐regulated by inflammatory stimuli, thus blocking the NF‐κB pathway upon stimulation and inactivating the host responses to bacterial infection. (B) GP2‐splicing variant 4 (GP2#4) rather than variant 2 induced by TNF‐α performs as a specific receptor for FimH‐positive bacteria to induce translocation of AIEC to the below Peyer's patches and activate protective immune responses. On the contrary, serum GP2 autoantibodies inhibit the FimH binding to GP2#4, thereby leading to a corresponding reinforced attachment of flagellated bacteria to other intestinal epithelium, impairing mucosal immune and exacerbating intestinal inflammation. (C) In healthy gut tissue, both human BCL‐G splice variants (BCL‐GL and BCL‐GS) are over‐expressed in IECs. In IBD patients, increased tissue expressions of Th1 cytokines (IFN‐γ and TNF‐α) can strongly suppress the BCL‐GS expression, which differentially regulates the inflammatory chemokines (CCL5 and CCL20). (D) In the PBMCs of IBD patients, both the full‐length and alternatively spliced variants (NOD2‐short and NOD2‐190) are synchronously down‐regulated, but only the former is active and responsive to the NF‐κB. (E) Eftud2 deletion in macrophage deregulates the mRNA splicing of MyD88/TLR4/MD‐2. Alternatively spliced forms of MyD88s, MD‐2B and sTLR4 may contribute to the inhibition of the TLR4 signalling, NF‐κB activation as well as the release of some pro‐inflammatory mediators. (F) The changes of different splice variants of IL23R mediate the STAT3 pathway via the ligand‐binding interaction.

New insights into the molecular mechanisms of IBD indicate that AS events can also induce an abnormal innate immunity and contribute to the risk of this disease, which have received a particular attention in recent years. Nucleotide‐binding oligomerisation domain 1 (NOD1), which contains three domains, including the C‐terminal region comprising various numbers of leucine‐rich repeat (LRR) domains, nucleotide‐binding domain and the N‐terminal caspase activation and recruitment domain (CARD), is an intracellular pattern recognition protein. Among them, the LRR domain plays a key role in achieving bacterial sensing. The downstream receptor‐interactional serine/threonine kinase (RICK), a caspase‐recruitment domain‐containing kinase, plays as a crucial medium of NOD1 and NOD2 signals. Once sensing the specific muropeptide, RICK can interact with the CARD of NOD protein and serves as a bridge between IκB kinase (IKK) and TGF‐β‐activated kinase 1 (TAK1) complex in the NF‐κB signalling pathway. Subsequently, TAK1 complex will activate the IKK complex, thus resulting in the NF‐κB activation and impairing the anti‐microbial response.^54^, ^55^, ^56^, ^57^, ^58^ In 2005, Girardin et al.^59^ identified the existence of several NOD1 splicing variants, and only the full‐length molecule could trigger the NF‐κB activation upon stimulation and activated the host responses to bacterial infection. In terms of IBD, three NOD1 splicing variants were up‐regulated by inflammatory stimuli, which could block the NF‐κB pathway induced by the full‐length molecule to favour the development of IBD (Figure 4A).^59^ In the last two decades, the adherent‐invasive *Escherichia coli* (AIEC) has been implicated in the pathophysiology of IBD. Adhesion of AIEC to IECs mainly depends on FimH and its binding receptor glycoprotein 2 (GP2) on the apical cell membrane of intestinal L or M cells.^60^, ^61^ An interesting study from Derer et al.^62^ reported that GP2‐splicing variant 4 (GP2#4) rather than variant 2 was specially expressed in intestinal L or M cells, which could be induced by TNF‐α. Initially, elevated GP2#4 performed as a particular receptor for FimH‐positive bacteria to induce translocation of AIEC to the below Peyer's patches and activate protective immune responses. Afterwards, the IBD‐related serum GP2 autoantibodies were generated and inhibited the FimH binding to GP2#4, thereby leading to a corresponding reinforced attachment of flagellated bacteria to other intestinal epithelium, impairing mucosal immune and exacerbating intestinal inflammation (Figure 4B).^62^ BCL‐Gonad (BCL‐G) is a unique and conserved member of the BCL‐2 family and initially considered as a pro‐apoptotic gene in humans.^63^ In healthy gut tissue, both human BCL‐G splice variants (BCL‐GL, long; BCL‐GS short) were found to be over‐expressed in IECs. Compared with the non‐IBD individuals, increased tissue expressions of Th1 cytokines (IFN‐γ and TNF‐α) could strongly suppress the BCL‐GS expression, which differentially regulated the inflammatory chemokines (CCL5 and CCL20) and thereby might drive the pathophysiology of IBD (Figure 4C).^64^

Similar to the function of NOD1, NOD2 is also identified as a crucial intracellular recognition receptor of pathogens, the ligand of which is muramyl dipeptide (MDP). Once bound to MDP, NOD2 can activate the NF‐κB signals, which brings out an up‐regulation of pro‐inflammatory cytokines.^65^, ^66^ In 2007, Leung et al. found that extensive AS targeting to the LRR domain and N‐terminal encoded the full‐length and alternatively spliced forms of NOD2 (NOD2‐short and NOD2‐190). In the peripheral blood mononuclear cells (PBMC) of IBD patients, both the full‐length and alternatively spliced variants were synchronously down‐regulated, but only the former was active and responsive to the NF‐κB. Thus, mutated variants of NOD2 might represent a novel mechanism in which the intracellular recognisation of bacterial peptidoglycan by the full length of NOD2 was significantly suppressed or altered (Figure 4D).^67^ Elongation factor Tu GTP binding domain containing 2 (Eftud2) is a crucial component of the U5 snRNP that modulates AS to possibly regulate innate immune response in C. elegans and mouse macrophage.^68^ Using an established mouse CAC model by azoxymethane (AOM)/dextran sulphate sodium (DSS), Lv et al.^69^ first demonstrated that Eftud2 was constantly over‐expressed in the colonic tissue samples as well as infiltrating macrophages. Oppositely, in myeloid‐specific Eftud2(−/−) mice, Eftud2 deletion in macrophages was found to deregulate the mRNA splicing of MyD88/TLR4/MD‐2. The alternatively spliced forms of MyD88s, MD‐2B and sTLR4 might contribute to the inhibition of toll‐like receptor 4 (TLR4) signalling, NF‐κB activation as well as the release of some pro‐inflammatory mediators (Figure 4E).^69^ Studies in recent years have identified the significance of IL‐23/IL‐23R signalling in regulating innate immune response by Th17 cells, and its downstream signal transducer and activator of transcription 3 (STAT3), janus‐kinase 2 and IL17RA have been also reported in IBD.^70^, ^71^ An interesting study from Kan et al.^72^ identified a series of newly spliced variants of IL23R, and discovered four diverse premature termination forms of IL‐23Ra. These changes might regulate the function of IL‐23R through influencing the ligand‐binding interaction and perhaps therefore represented an inherent protective mechanism against the pathogenesis of IBD (Figure 4F).^72^

### 2.4 AS and adaptive immune

**FIGURE 5:**
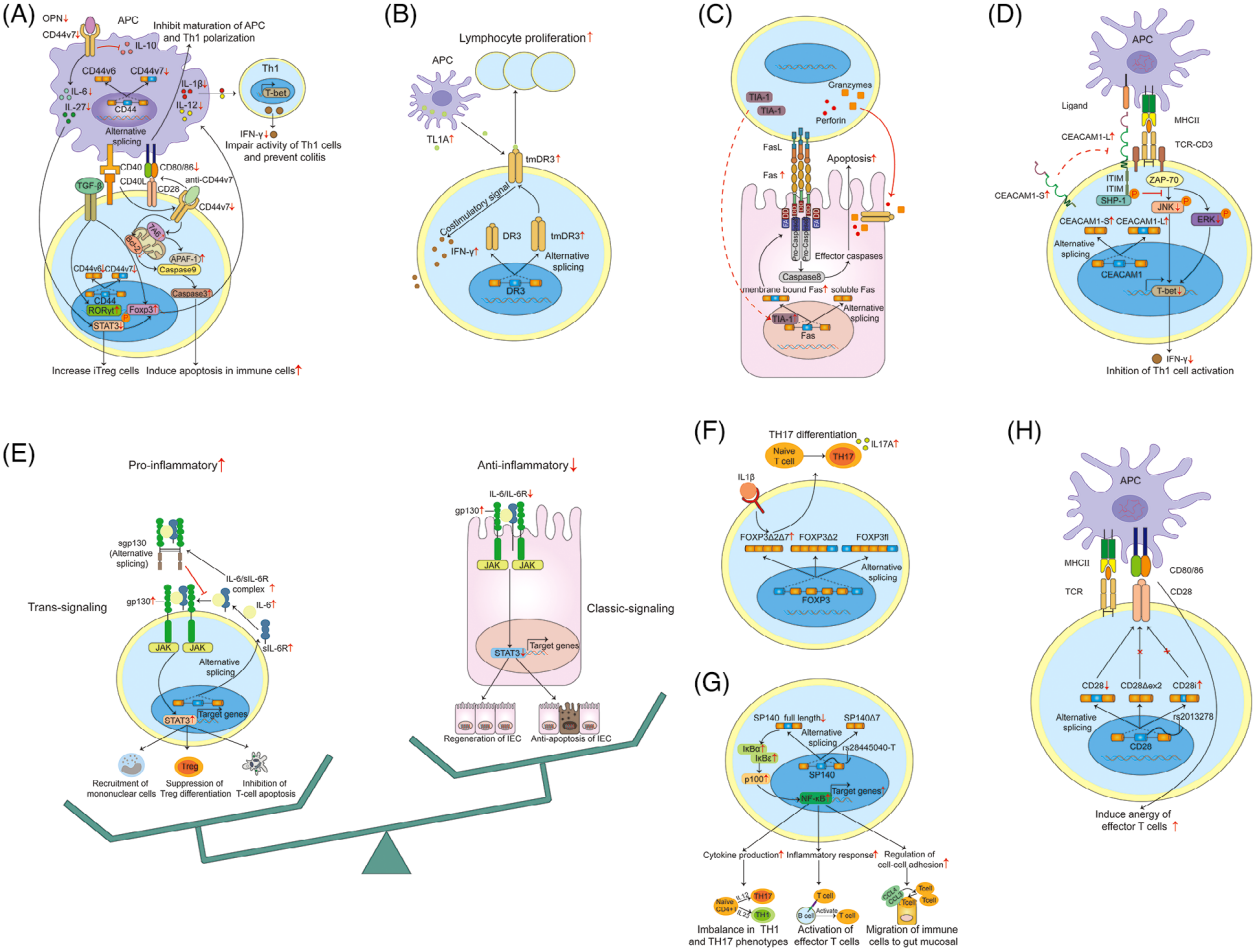
AS and adaptive immune. (A) In intestinal macrophage and T cells, blockade of CD44v6 and CD44v7 induces apoptosis in immune cells and prevent the chronic inflammation by distinct pathways. (B) Predominant expression of the tmDR3 in preference to the soluble form on lymphocytes can trigger the costimulatory signals, amplify the IFN‐γ secretion and connect to the pathogenesis of Th1‐associated inflammation. (C) In CTLs, TIA‐1 acts as an AS regulator, and generates a membrane‐bound form of FAS receptor, which can lead to more cryptal apoptosis and abscesses. (D) In mouse intestinal T cells, owing to the two phosphorylated ITIMs, up‐regulated CEACAM1‐L and CEACAM1‐S after ligand interaction mediate the TCR–CD3 pathway, and result in the inhibition of Th1 differentiation and secretion. (E) Classic IL‐6 activation via IL‐6Rs seems to play a protective role, while sIL‐6R‐mediated cell signal (IL‐6 trans‐signalling) exerts pro‐inflammatory effects. (F) IL‐1β promotes abnormal patterns of FOXP3 splicing with an up‐regulated proportion of FOXP3Δ2Δ7, which can favour the differentiation of naïve T cells into Th17 cells, and contribute to IL‐17 production and disease severity. (G) SP140Δ7 altered by rs28445040‐T inhibits the NF‐κB activity in B cells, and is involved in regulation of cytokine production, inflammatory response and cell‐cell adhesion. (H) Both CD28i and CD28Δex2 associated with ligands are confirmed as loss‐of‐function splicing isoform products that can reduce disease risk by inducing anergy of effector T cells.

In contrast to the innate immunity, adaptive immune responses involved in the IBD development are more time‐consuming, precise and complex. Once initiated by signals from microorganisms and damaged tissue, antigen‐presenting cells (APC) present antigens to T or B lymphocytes, and aggressive T or B cells via their productions of IgG antibodies will initiate a state of chronic inflammation response.^73^, ^74^ Recently, immunologists have also observed that AS events may participate into the different steps of adaptive immune response in IBD, such as activating the Th lymphocytes (Th1, Th2, Th17 and Th22 cells) and suppressing the activity of regulatory T (Treg) cells. CD44 is a widely expressed cell surface glycoprotein and transmembrane adhesion molecule that functions in many processes such as haematopoiesis, lymphocyte activation and tumour progression. Diverse CD44 isoforms are generated from AS of up to 10 separate exons (v1–v10).^75^, ^76^ As early as 1995 and 1996, the descriptions of alternatively spliced CD44 species were proposed between the normal, inflammatory and neoplastic lesions by Rosenberg and Yoshida, respectively, which first unravelled the mystery of CD44 variants in IBD patients.^77^, ^78^ In the study of Rosenberg et al.,^77^ the epithelial expressions of CD44v3 and CD44v6 were found to be significantly up‐regulated in biopsy samples of UC patients, but not in colonic CD patients. The possible mechanisms might be that these two CD44 isoforms (CD44v3 and CD44V6) could increase the lamina propria lymphocyte adhesion in colonic tissues of UC patients.^79^, ^80^ Similarly, the up‐regulation of CD44v6 was also confirmed in the lesions of inflamed IBD colonic epithelium as a means of assessing the disease activity,^81^ whereas another report from Pfister et al.^82^ found that CD44v6 was deceased on CD4^+^ lamina propria T cells in the mucosa of IBD patients. At the same time, another interesting variant CD44v7 began to be discovered in autoimmune disease and IBD.^83^, ^84^ On the one hand, CD44v7 appeared to endow lamina propria mononuclear cells with downstream contra‐apoptotic signals that might led to resistance to apoptosis and sustenance of the chronic colitis.^85^ On the other hand, the expression of CD44v7 isoform on macrophages was proved to be indispensable for provoking the chronic colonic inflammation in the mice gut. CD44v7 deletion in macrophages of recipient mice might link to the down‐regulation of STAT3‐activating and forkhead box P3 (Foxp3)‐counteracting IL‐6, which would cause decreased numbers of phospho‐STAT3‐containing lymphocytes as well as elevated counts of Foxp3^+^ T‐cells in the gut (Figure 5A).^86^ TL1A as well as its functional receptor (death‐domain receptor 3, DR3) have been considered as the key members of TNF/tumour necrosis factor receptor superfamilies of proteins. Once APC‐derived TL1A is bound to the lymphocytic DR3, TL1A–DR3 interaction exerts pleiotropic effects on different adaptive immune cells, including Treg and helper T cells, to influence cell proliferation, maintenance and differentiation.^87^, ^88^ It was confirmed that chronic colonic inflammation linked to the AS of DR3. Predominant expression of the transmembrane form of the receptor DR3 (tmDR3) in preference to the soluble form on lymphocytes could trigger the costimulatory signals that significantly amplified the IFN‐γ secretion and connected to the pathogenesis of Th1‐associated inflammation (Figure 5B).^89^ In normal physiologically relevant conditions, T lymphocytes exist in the intestine epithelium (also known as intraepithelial lymphocytes, IELs). In cases of IBD, evidence shows that cytotoxic T lymphocytes are present in increased numbers and can promote the cryptal apoptosis and mucosal damage by releasing cytotoxic products, including granzymes and perforin, and the interaction of Fas ligand (Fas L) with a transmembrane death‐signalling receptor Fas.^90^, ^91^ Two studies in 2000 and 2001 all proved that T cell‐restricted intracellular antigen (TIA‐1) might serve on a mediator of alternative pre‐mRNA splicing, and could generate a mRNA isoform that coded for the membrane‐bound form of FAS receptor.^92^, ^93^ By immunohistochemical analyses from the IBD mucosal biopsy specimens and normal controls, Mitomi et al.^91^ demonstrated that TIA‐1 + IELs were significantly elevated as compared with healthy individuals, and thereby leading to more cryptal apoptosis and abscesses in the destructive inflammatory condition of IBD (Figure 5C). Carcinoembryonic antigen‐related cell adhesion molecule 1 (CEACAM1), that is belonged to the carcinoembryonic antigen (CEA) family, is considered as a cell‐cell adhesion receptor and holds a complex role in inflammation and tumours.^94^, ^95^ The alternative CEACAM1 variants in human differ in the length of cytoplasmic tail and variable membrane distant Ig‐like domains. The long variants contain two intracellular immunoreceptor tyrosine‐based inhibitory motifs (ITIM) that negatively regulate the T cell activation, while the short isoforms lack the ITIM sequences that serves as costimulatory receptors.^96^ In IBD, CEACAM1 was confirmed as a newly, non‐CD28‐associated co‐inhibitory receptor that mediated suppression of the T‐cell receptor–CD3 complex in a cell autonomous manner, and subsequently blocked the progression of intestinal inflammation.^97^ In 2006, in the murine colitis model, Nagaishi et al.^96^ revealed that over‐expression of the CEACAM1 isoform (CEACAM1‐4L) specifically in T lymphocytes might result in T‐cell inhibition ex vivo. In the study of Chen et al., CEACAM1‐L and CEACAM1‐S, were found to be up‐regulated after the protein–protein interaction. Owing to the two phosphorylated ITIMs, CEACAM1‐L could inhibit TCR–CD3 complex induced signal cascade, which depended upon the Src homology domain phosphatases 1 (SHP‐1) activity. Then the inhibitory role of CEACAM1‐L was mediated by the phosphorylation of C‐Jun N‐terminal kinase (JNK) and extracellular response kinase (ERK) pathways, thus leading to inhibition of Th1 pathways and cytokines secretion.^95^, ^96^, ^98^ (Figure 5D). Interleukin‐6 (IL‐6) is a both pro‐ and anti‐inflammatory mediator and exerts multiple functions when largely induced during infection, inflammation and cancer.^99^ A growing body of evidence show that two models of IL‐6 activation are presented and plays a dual role in the process of IBD as well as CAC. On one hand, the classic IL‐6 activation through membrane‐bound IL‐6 receptors (IL‐6Rs) seems to play a protective role that the regenerative and anti‐apoptotic response of IECs to the damage induced by DSS are observed. On the other hand, a soluble IL‐6R (sIL‐6R)‐conducted cell signal (IL‐6 trans‐signalling) promotes pro‐inflammatory pathway by activating the immune systems, including recruiting mononuclear cells, inhibiting T‐cell apoptosis and suppressing Treg differentiation.^100^, ^101^, ^102^, ^103^, ^104^, ^105^ It is interesting that two types of IL‐6 receptor subunit (IL‐6R or sIL‐6R; gp130 or sgp130) are also generated by differential corresponding pre‐mRNA splicing.^106^, ^107^ Recently, it was demonstrated that blockade of IL‐6 trans‐signalling by anti‐IL‐6R antibodies or recombinant sgp130 protein bound to the Fc region of human IgG1 might not only partly ameliorate the development of IBD but also the CAC progression (Figure 5E).^108^ Treg cells are key regulators of inflammation and as well as in the maintenance of immune tolerance and homeostasis.^109^ Forkhead box P3 (FOXP3, as a primary transcription factor of Treg cells) is necessary for the development of Treg cells. AS events consequently allow a single FOXP3 gene to produce different isoforms, including FOXP3fl (the full‐length FOXP3), FOXP3Δ2 (FOXP3 lacking exon 2) and FOXP3Δ2Δ7 (FOXP3 lacking exon 2 and 7), which exert multiple or even opposing functions.^110^, ^111^ In patients suffering CD, the pro‐inflammatory cytokine IL‐1β was found to promote abnormal patterns of FOXP3 splicing with an elevated proportion of FOXP3Δ2Δ7, which could favour the differentiation of naïve T cells into Th17 cells, and contribute to IL‐17 production and disease severity (Figure 5F).^112^ Speckled Protein 140 (SP140) is a nuclear protein that is belonged to the speckled protein (SP) family, and its loss‐of‐function mutations is associated with multiple sclerosis (MS), CD and chronic lymphocytic leukaemia.^113^, ^114^ A study from Fraschilla et al.^115^ demonstrated that Sp140^−/−^ mice might harbour altered microbiota and exhibit more severe colitis. Mechanistically, a causal variant of rs28445040‐T was found to alter the splicing of the 7th exon of SP140 gene, which produced a transcript lacking the exon 7 (SP140Δ7) and decreased the full‐length transcript expression. These changes subsequently reduced the SP140 protein expression in lymphoblastoid cells and inhibited the NF‐κB activity in B cells. GO analysis implied that differentially expressed genes (DEGs) after SP140 silencing were enriched in regulation of inflammatory response, cell–cell adhesion and cytokine production, thereby leading to the progression of IBD (Figure 5G).^116^ CD28 is a 44‐kDa homodimeric glycoprotein expressed on the surface of the majority of T cells. Previous data have revealed that CD28 is a key co‐stimulatory molecular, and ligation of CD28 with ligands (CD80/CD86) may play an essential role in naïve T cell activation.^117^ In 2022, the existence of AS isoforms of CD28 was reported. Among these IBD‐related isoforms, full‐length CD28 was demonstrated to show a higher binding affinity with CD80/CD86, while both CD28i and CD28Δex2 were confirmed as loss‐of‐function spliced products that might decrease the disease risk through bringing out anergy of effector T cells, inducing tolerance, and inactivation to intestinal antigens and allergens (Figure 5H).^118^

### 2.5 AS and fibrosis

**FIGURE 6:**
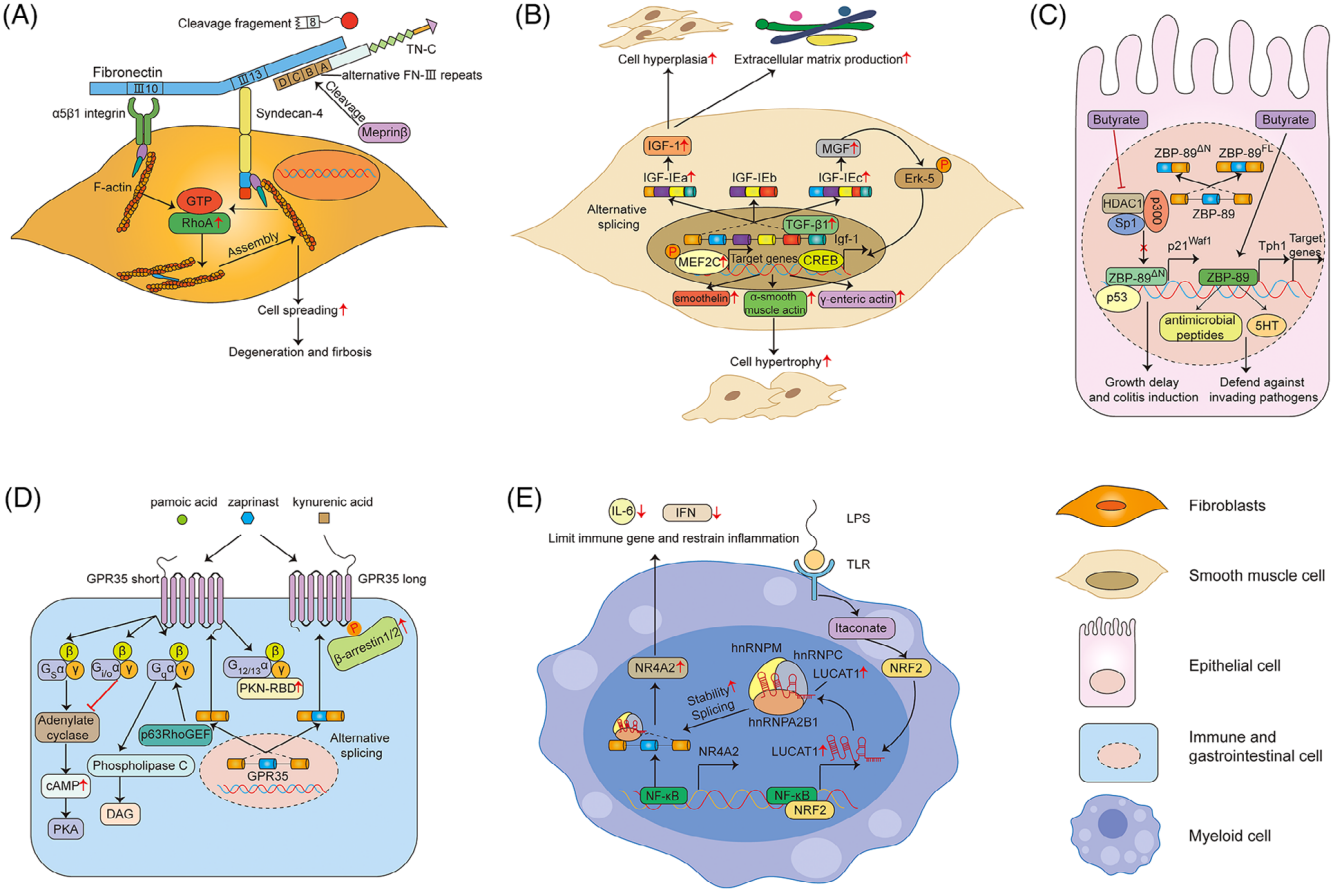
AS regulates fibrosis and other risk factors in the pathogenesis of IBD. (A)TN‐C that contains an alternatively spliced FNIII repeats A1–D interferes the FN/syndecan‐4 interaction, resulting in actin stress fibre assembly and cell spreading. (B) The splicing isoforms of IGF‐I induced by TGF‐1 are involved in cell hyperplasia, cell hypertrophy and extracellular matrix production in smooth muscle cell. (C) Two ZBP‐89 splice isoforms (ZBP‐89FL and ZBP‐89DN) regulate biological functions in epithelial cells. ZBP‐89^FL^ tends to protect against chronic colitis, while ZBP‐89^DN^ renders the colonic mucosa more susceptible to colitis. (D) Two distinct variants of GPR35 (GPR35 long and short) implicate in gut‐related diseases, and these two variants differed only in the length of their extracellular N‐termini by 31 amino acids. The short isoform can activate different major G proteins, while the presence of GRP35 long plays a positive modulator for arrestin recruitment. (E) LUCAT1 controls the splicing and stability of anti‐inflammatory NR4A2, thereby contributing to the suppressing effects of interferons and inflammatory mediators.

Chronic inflammation is a prerequisite for CD, but progression to strictures is predominantly driven by intestinal fibrosis. Investigations on the mechanism of fibrosis involve the excessive accumulation of extracellular matrix and expansion of mesenchymal cells, such as myofibroblasts, fibroblasts and smooth muscle cells.^119^, ^120^, ^121^ Fibroblasts synergistically bind to fibronectin (FN) through integrin α5β1 and syndecan‐4 (recognising the 10th FN‐III domain (III10) and the 13th FN‐III domain (III13) of FN, respectively), which can activate the RhoA signals, and induce the assembly of actin stress fibre and cell spreading.^122^, ^123^ Tenascin‐C (TN‐C), which is a extracellular matrix glycoprotein and contains an alternatively spliced FNIII repeats A1‐D, can presumably bind to the 13th FN‐III domain and syndecan‐4, interfere with the FN/syndecan‐4 interaction.^122^, ^124^ In CD patients, though TN‐C was significantly induced in inflamed lesions of the colonic mucosa, many meprinβ‐positive leukocytes that appeared throughout the aberrant and inflamed intestinal tissue was able to cut the spliced N‐terminal of TN‐C at two distinct cleavage sites (the 7th constant FN‐III repeat and the alternative FN‐III repeat D). For this reason, the reactivated RhoA signalling might partly drive the FN‐mediated sustained fibroblasts activation, thereby progression of fibrosis in the pathogenesis of CD (Figure 6A).^125^ Insulin‐like growth factor‐I (IGF‐I) that is induced by the fibrogenic cytokine TGF‐1, is highly expressed in all layers of intestinal in CD patients.^126^ This gene can be alternatively spliced into IGF‐IEa, IGF‐IEb and IGF‐IEc variants in humans. Among these variants, the IGF‐IEa splice variant was confirmed to encode pro‐IGF‐IEa producing mature IGF‐I, result in smooth muscle hyperplasia and excessive collagen I productions.^127^ Simultaneously, another up‐regulated variant IGF‐IEc might significantly increase the phosphorylated levels of myocyte enhancer factor 2 C (MEF2C) and extracellular signal‐related kinase (Erk5), which controlled the transcription of smooth muscle‐particular proteins, such as smoothelin, α‐smooth muscle actin and γ‐smooth muscle actin. These elevated molecules were all essential participants in the smooth muscle cell hypertrophy, which contributed to the formation of intestinal stricture (Figure 6B).^128^

### 2.6 AS and other risk factors

In addition to the potential mechanisms discussed above, recent investigations also have demonstrated that AS events indeed participate into the IBD development in several different manners. For instance, ZBP‐89 (Zfp148, ZNF148, BFCOL1, BERF1), is a zinc finger transcription factor, that can bind to GC‐rich DNA elements in promoters, and involves the cell growth and death regulation. Two ZBP‐89 splice isoforms (ZBP‐89^FL^ and ZBP‐89^DN^) have been identified and co‐expressed in gastrointestinal cell lines and tissues, which may regulate diverse biological functions in colitis. ZBP‐89^FL^ was found to directly bind to the TPH1 promoter, which encoded the rate‐limiting enzyme in 5HT biosynthesis, and subsequently generated optimal amounts of 5HT or other antimicrobial peptides in response to bacterial infections. This data implied that the expression of ZBP‐89^FL^ tended to protect against chronic colitis.^129^, ^130^ The other ZBP‐89 spliced isoform (ZBP‐89^DN^), which retained its zinc‐finger domain, could interact directly with p53 protein, while loss of amino terminal residues 1−127 of the full‐length protein might interfere the butyrate‐mediated p21waf1 activation by interacting with p300. As a consequence, ZBP‐89^DN/DN^ mice might experience growth delay, decreased viability and rendered the colonic mucosa more susceptible to DSS colitis (Figure 6C).^131^ Emerging evidence shows that the mechanisms that G protein‐coupled receptor 35 (GPR35) modulates the pathological processes of gastrointestinal inflammation through the mucosal healing of colonic epithelium,^132^ immune system^133^ and intestinal homeostasis.^134^, ^135^ GPR35, is a receptor for lysophosphatidic acid, and over‐expressed in IECs and some specific subtypes of immune cells. Single‐nucleotide polymorphisms also confirmed GPR35 as a key susceptibility gene for IBD.^136^ Recently, investigators also elaborated on the two distinct variants of GPR35 (GPR35 long and short) implicated in gut‐related diseases, and these two variants differed only in the length of their extracellular N‐termini by 31 amino acids. The short isoform could activate different major G proteins, while the presence of GRP35 long played a positive modulator for arrestin recruitment (Figure 6D).^137^ As mentioned above, FN is an adhesive glycoprotein existing in the extracellular matrix, and the heterogeneity of FN subunits derives mainly from AS of a primary precursor mRNA at three distinct sites termed EDB, EDA and IIICS.^138^, ^139^ In 2015, Bootz et al.^140^ found that the alternatively spliced EDA domain of FN could not be virtually detected in most adult normal organs, while it was strongly stained in the sub‐mucosa and certain structures in the muscularis mucosa around blood vessels within the specimens of IBD patients and mice models of colitis. This means that exploring specific antibodies to the EDA domain of FN may provide us a prospective therapeutical option for the treatment of IBD conditions.^140^ It is known that glucocorticoids (GC) exert an established immunosuppressive effect and are widely applied in the moderate‐to‐severe IBD treatment. The response to GC is mainly mediated through glucocorticoid receptor (hGR), of which two isoforms hGRα and hGRβ exist.^141^, ^142^ In 2005, Towers et al.^143^ evaluated the hGRα and hGRβ expressions in CD patients and looked for a potential link between these two receptors and their response to GC treatment. The data implied that the over‐expression of hGRα mRNA in active CD patients was independent on steroid‐resistant or steroid‐responsive. But the augmented expressions of hGRβ were connected with GC resistant during the active phase of UC and CD patients.^143^ However, in 2007 Hausmann and coworkers published the controversial data that neither of the GC isoforms associated with the GC sensitivity, which denied its predictive value for efficacy of steroid treatment.^144^

In addition to the molecules mentioned above in which their potential regulatory mechanisms in the pathogenesis of IBD have been fully elucidated, there are still many well‐established positive or negative regulatory genes encoded by different variants but lacking of detailed elaborations for their possible pro‐ or anti‐inflammatory mechanisms. For example, some newly reported molecules, such as caspase‐associated recruitment domain 8 (CARD8), neurokinin‐1 receptor (NK‐1R), orosomucoid 1‐like protein 3 (ORMDL3), protein tyrosine phosphatase non‐receptor type 2 (PTPN2), IL‐15 receptor alpha (IL‐15Rα) are all positional and functional candidate genes for IBD, and variants of splicing of these genes have been addressed by numerous reports.^145^, ^146^, ^147^, ^148^, ^149^ NK‐1R, a principal receptor of pro‐inflammatory neuropeptide substance P (SP), has been proved to play a vital role in rodent models of chronic colitis and in UC as well as CD patients.^150^, ^151^ In 2015, Gillespie et al. first examined the truncated (tr‐NK‐1R) and full‐length (fl‐NK‐1R) receptor expressions in colonic tissues from patients of quiescent colitis, high‐grade dysplasia (HGD) and CAC. The data implied that enhance of total NK‐1R protein in HGD and CAC was attributable to an elevation of tr‐NK‐1R mRNA, strongly suggesting an essential role of tr‐NK‐1R during the colitis‐to‐CAC malignant transformation. The tr‐NK‐1R variant could be therefore served as a diagnostic biomarker to distinguish patients at risk of neoplasia or associated cancer.^146^ Nuclear receptor subfamily 4, group A, member 2 (NR4A2) is a nuclear receptor involving in modulating target gene transcription and regulating distinctive physiological processes.^152^, ^153^ In IBD, NR4A2 was considered as a negative regulator of immune response, and deletion of NR4A2 in T cells was confirmed to attenuate Tregs induction and led to aberrant increase of Th1 cells, which might partly exacerbate the colonic inflammation.^154^ Likewise, NR4A2 is also a spliced gene,^155^, ^156^ and a recent study from Vierbuchen identified NR4A2 as a downstream mediator and binding protein of nuclear long non‐coding RNA LUCAT1‐dependent immune gene suppression. LUCAT1 was induced to control the splicing and stability of anti‐inflammatory NR4A2, thereby contributing to the suppressing effects of interferons and inflammatory mediators (Figure 6E).^157^

## 3 TRANSCRIPTOMIC ANALYSIS OF AS EVENTS IN THE PATHOGENESIS OF IBD

**FIGURE 7:**
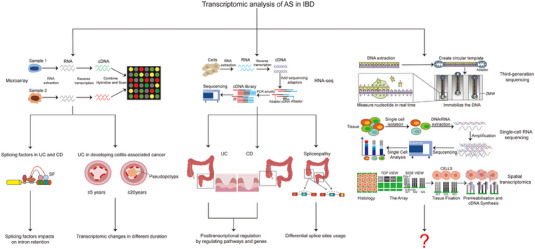
Transcriptomic analysis of AS events in the pathogenesis of IBD. Microarrays and NGS have emerged as powerful approaches that provide investigators highly sensitive and robust examinations for AS events in IBD. Notably, newly technologies, such as TGS, scRNA‐seq and ST, are also desired with great expectations.

As described above, we have systematically summarised all single splicing molecules that may cause AS events in the pathogenesis of IBD within the last two decades. However, we shall take a comprehensive and profound view of this problem. The main reason is that both UC and CD are highly complex disease processes that result from the integration of multiple and incompletely identified pathogenic elements. Therefore, transcriptomics has emerged as a powerful approach that provides us a highly sensitive and robust examination for multiple attributes such as strandedness, sequence composition, splicing factors, AS and alternative transcription start/stop sites (Figure 7).^158^ Since the introduction of microarrays as the mainstream technology of the last decade, researchers have provided dozens of valuable datasets for identifying DEGs in large IBD samples readily available.^159^, ^160^ In 2011, Häsler et al.^161^ first used the commercial and custom‐designed microarrays to investigate the gene expressing profile of pre‐mRNA splicing factors, and identified intron retention as an exemplary splicing event that was possibly linked to the IBD aetiology. A number of population studies have demonstrated that patients with long duration of UC present a relatively higher risk of developing CAC, in comparison with those patients with a shorter‐time inflammation.^162^, ^163^ One of proposed mechanisms may due to accumulated genetic abnormalities when IECs are persistently and chronically exposed to long‐term inflammation.^164^ From this, using the Affymetrix Human Transcriptome Array 2.0, a similar study from the Asia Pacific region had been conducted to screen the transcriptome profiling, including DEGs and AS events, in inflamed colonic biopsies of long‐ and short‐duration UC patients. Totally, 640 DEGs and 3560 genes with differential splicing were identified.^165^

RNA‐seq, the current next‐generation sequencing (NGS) approach, is expected to a superior technology to microarrays, which can provide global and digital rather than analogue information on transcripts and their corresponding isoforms. In the last years, it has merged as a revolutionising and mainstream approach and used to interrogate the transcriptome in various illnesses, as well as some chronic immune‐mediated disorders.^166^, ^167^, ^168^ From this, genome‐wide analyses of gene expression alternations and AS signatures in patients with different subtypes of IBD are also performed by RNA‐seq. For example, a recent pioneering study by Li et al. presented an interesting genomic landscape of AS signatures in UC patients based on RNA‐seq data from two cohorts, and found that skipped exon (SE) and alternative first exon (AFE) were the two most remarkably enriched AS events during the UC development. In addition, they also performed a combined mRNA‐seq experiment between four UC patients and four healthy individuals and discovered that the immune response‐associated pathways and cell chemotaxis were significantly enriched in UC‐related AS events.^169^ Coincidentally, another study from the same research team employing a well‐established public NCBI GEO dataset (GSE66207) identified a total of 2980 important AS events in CD patients, in comparison with controls. To validate the reliability in the GSE66207 dataset, authors also analysed the RNA‐seq data focused on a Chinese cohort and demonstrated 1715 significantly AS events were involved. Interestingly, the results from public or validation RNA‐seq dataset all suggested a strong similarity that SE and AFE were the two most common types of AS events in patients with CD.^170^ In 2021, a transcriptomic research using RNA‐seq was carried out to present the whole mRNA sequencing profiles of 124 biopsies obtained from 34 young donors with UC or CD. In this study, a newly definition of ‘spliceopathy’ was first supported by Berger et al.,^171^ and the meaningful results implied that tissue location might be the largest contributor to variability in gene expression and splicing of IBD patients.

## 4 FUTURE PERSPECTIVES

With research progresses, many investigators have now noticed the significance of abnormal AS events accompanied by the occurrence and progression of diverse diseases, which are able to produce multiple different isoforms and diversify protein products. For instance, our recent published data using the long‐read sequencing technology first investigated the potential spicing events in CRC. Among the newly identified splicing isoforms, tissue inhibitor of metalloproteinase‐1 Δ4‐5 transcript (TIMP Δ4‐5) was significantly down‐regulated in CRC tissues, while the full length of TIMP (TIMP‐FL) possibly served as an oncogenic transcript and promoted the CRC growth.^172^ Recently, AS investigations has also gained considerable momentum during the initiation and development of IBD. Though this study has described the regulatory mechanisms of AS, as well as its detection in patients with IBD and CAC, there are still some relative limitations. First, in addition to coding RNAs, non‐coding RNAs, including circular RNAs, microRNAs, long non‐coding RNAs and small nuclear RNAs, are also inextricably linked to AS.^173^, ^174^, ^175^ These multiple different types of ncRNAs, which are generated by AS of precursor messenger RNAs, have been also implicated in the initiation, progression and therapy resistance or acted as regulatory molecules in various types of diseases through AS.^176^, ^177^ But it is a pity that there are not still any reports focusing on the non‐coding RNAs through AS events to elucidate the occurrence of IBD. Second, the majority of literature mainly reported the novel splicing molecules or elucidate the potential mechanisms of AS in IBD patients or experimental models, whereas no validated AS‐related biomarkers of IBD have been fully investigated or used in clinic. Third, the revolution of genome‐wide analyses of gene expression alternations and AS events is continuing after the successful applications of microarrays and NGS technology. Third‐generation sequencing (TGS) is proved to be a newly and improved sequencing technology, which can access in‐depth the splicing regulation, enhance the RNA isoforms’ characterisation and predict more comprehensive gene expression diversity.^178^, ^179^ In addition, traditional methods determine the DEGs owing to the analyses of whole‐tissue samples, but the contribution of individual cell populations are unknown. Single‐cell RNA sequencing (scRNA‐seq) including genomics, transcriptomics, proteomics, epigenomics and metabolomics sequencing, can successfully resolve this problem and directly measure molecular signatures in thousands to millions of individual cells, which provides us an opportunity to uncover the mysteries underlying cellular populations.^180^ Since its introduction in 2009,^181^ studies based on scRNA‐seq are rapidly increasing and have discovered more and more profound information about health and diseases.^182^, ^183^ The scRNA‐seq technology is also employed by many investigators to characterise cell‐type‐specific transcriptional heterogeneity in IBD.^184^, ^185^, ^186^, ^187^ Besides, a prevailing ‘spatial transcriptomics’ (ST) has been developed that allows spatially resolved, high‐dimensional assessment of gene transcription. Researches are able to obtain more high‐quality RNA‐sequencing data with three‐dimensional positional information from tissues sections.^182^, ^188^, ^189^ More interestingly, scRNA‐seq and ST are not contradictory. On the contrary, these two current technologies can be integrated, and newly integrative computational methods are also studied in depth to propose ways to effectively capture more useful signatures in biomedical researches.^190^, ^191^, ^192^, ^193^ These three newly developed technologies can help investigators better understand the complex pathogenesis of IBD. It is believed that these currently developed technologies have opened a door for us to broaden the scope of the complex IBD pathogenesis, and more significant IBD‐related AS events and variants will be revealed and studied in the near future (Figure 7) . At last, this review presents all current data concerning the pathogenic role of AS during the IBD progression (Table 1). This may help us to further understand the molecular mechanisms of IBD, and develop and find new therapeutic methods or targets for IBD treatment, such as the development of an IL‐6R‐specific mAb, optimised version of sgp130Fc, some other small‐molecule splicing modulators and splice site‐switching anti‐sense oligonucleotides.^104^, ^194^ Of course, though great efforts have been made, further studies are still required to address the associations between AS and IBD in more detail.

**TABLE 1:** Overview of AS‐related genes and variants modulated in IBD.

Gene	Locus	Spliceosome	Splice variant	Sample	Source	Up/Down‐regulation	Function	References
MYLK	3q21.1	N/A	MLCK1 MLCK2	Colonic mucosal samples with UC and CD, DSS‐induced model sample	Human and mice	Up‐regulation	Pro‐inflammatory	^39^
KAT2A	17q21.2	SF3b complex	KAT2A	Colonic biopsies from patients with IBD and CRC	Human	Up‐regulation	Pro‐inflammatory	^40^
LmnA	1q22	U2 and SR	Progerin	Colonic biopsies from healthy controls and patients with IBD, Cbx3 mouse model	Human and mice	Up‐regulation	Pro‐inflammatory	^41^
PTPRS	19p13.3	N/A	Isoform2 Isoform3 Novel isoform1	DSS‐induced model sample	Mice	Up‐regulation	Pro‐inflammatory	^47^
KIND1	20p12.3	N/A	Kindlin‐1 short	Intestinal biopsies from KS patients	Human	Up‐regulation	Pro‐inflammatory	^48^
MYPT1	12q21.2‐q21.31	N/A	MYPT1 variant2	Colonic biopsies from mouse sample	Mice	N/A	Pro‐inflammatory	^50^
GPR137	11q13.1	ESRP1	Gpr137‐short Gpr137‐long	Tumour, adenoma and normal tissue from CRC patient, AOM/DSS model sample	Human and mice	Up‐regulation	Pro‐inflammatory	^51^
NOD1	7p14.3	N/A	Nod1Δ10 Nod1Δ10‐11 Nod1Δ10‐12	Human epithelial cells	Human	Up‐regulation	Pro‐inflammatory	^59^
GP‐2	16p12.3	N/A	GP2#2 GP2#4	Ileal and colonic biopsies from IBD patients	Human	Up‐regulation	Pro‐inflammatory	^62^
BCL‐G	12p13.2	N/A	BCL‐Gs BCL‐GL	Colonic biopsies from non‐IBD and IBD patients	Human	Down‐regulation	Pro‐inflammatory	^64^
NOD2	16q12.1	N/A	NOD2‐190 NOD2‐short	Peripheral blood mononuclear cells (PBMC) from healthy donors	Human	Up‐regulation	Pro‐inflammatory	^67^
MyD88 TLR4 MD‐2	3p22.2 9q33.1 8q21.11	Eftud2	MyD88s sTLR4 MD‐2s	DSS‐induced model sample	Mice	Up‐regulation	Anti‐inflammatory	^69^
IL23R	1p31.3	N/A	Short peptide Soluble form Truncated Extracellular Non‐responsive	PBMC from healthy donors	Human	Down‐regulation	Anti‐inflammatory	^72^
CD44	11p13	N/A	CD44v7 CD44v6	Transgenic mice model, colonic biopsies from patient with IBD,	Human and mice	Down‐regulation	Anti‐inflammatory	^84^, ^86^
DR3	1p36.31	N/A	tmDR3	Ileitis murine model	Mice	Up‐regulation	Pro‐inflammatory	^89^
Fas	10q23.31	N/A	Membrane bound Fas	Colonic biopsies from patient with IBD	Human	Up‐regulation	Anti‐inflammatory	^91^
CEACAM1	19q13.2	N/A	CEACAM1‐L CEACAM1‐S	Jurkat‐T cell transfection model	Human	Up‐regulation	Anti‐inflammatory	^96^
IL‐6R	1q21.3	N/A	sIL‐6R	AOM/DSS‐induced model sample	Mice	Up‐regulation	Pro‐inflammatory	^100^, ^101^, ^102^, ^103^, ^104^, ^105^, ^106^, ^107^, ^108^
FOXP3	Xp11.23	N/A	FOXP3Δ2 FOXP3Δ2Δ7	Colonic biopsies from CD patients	Human	Up‐regulation	Pro‐inflammatory	^112^
SP140	2q37.1	N/A	SP140Δ7	Lymphoblastoid cell line	Fetal bovine	Down‐regulation	Pro‐inflammatory	^116^
CD28	2q33.2	N/A	CD28Δex2 CD28i	Jurkat‐T cell transfection model		Up‐regulation	Anti‐inflammatory	^118^
TN‐C	9q33.1	N/A	FNIIID	Transfected plasmid, chicken embryo	Chicken	N/A	Fibrosis	^125^
Igf‐1	12q23.2	N/A	IGF‐IEa IGF‐IEb IGF‐IEc	Intestinal muscle cell from patient with CD and healthy donors	Human	Up‐regulation	Cell hyperplasia, hypertrophy	^128^
ZBP‐89	3q21.2	N/A	ZBP‐89^ΔN^ ZBP‐89^FL^	DSS‐induced model sample	Mice	Up‐regulation	Pro‐inflammatory	^131^
GPR35	2q37.3	N/A	GPR35 short GPR35 long	Transfected plasmid	N/A	N/A	Mediate intracellular pathways	^137^
EDA	Xq13.1	N/A	EDA	DSS‐induced model sample	Mice	Up‐regulation	Pro‐inflammatory	^140^
GR	5q31.3	N/A	GRα GRβ	Colonic tissue from CD patients and healthy donors	Human	Up‐regulation	Steroid resistance	^143^
CARD8	19q13.33	N/A	T60, T54, T51, T48, T47	Lymphoblastoid cell lines of CD patients	Human	N/A	N/A	^145^
NK‐1R	2p12	N/A	tr‐NK‐1R fl‐NK‐1R	Colonic tissue from UC patients	Human	Up‐regulation	Malignant transformation	^146^
ORMDL3	17q21.1	N/A	ORMDL3 V1	Hela cells, HEK293 cells, HL60 cells	Human	N/A	N/A	^147^
PTPN2	18q11.21	N/A	PTPN2‐001 PTPN2‐002 PTPN2‐003	Human peripheral blood mononuclear cells and mouse peritoneal macrophages	Human and mice	N/A	N/A	^148^
IL15RA	10p15.1	N/A	Variant1 Variant2 Variant3	Colon and duodenum biopsy specimens from healthy individuals	Human	N/A	N/A	^149^
NR4A2	2q24.1	hnRNPC hnRNPM hnRNPA2B1	N/A	PBMC from donors	Human	Up‐regulation	Anti‐inflammatory	^157^

## AUTHOR CONTRIBUTIONS

Chunfang Xu, Airong Wu and Qiaoming Zhi designed the study. Chentao Zou, Xinquan Zan and Zhenyu Jia collected all literature and wrote the manuscript. Lu Zheng, Yijie Gu, Fei Liu and Ye Han provided the technical and writing supports. All authors read and approved the final manuscript.

## CONFLICT OF INTEREST STATEMENT

The authors declare that they have no conflict of interest.

## ETHICS APPROVAL AND CONSENT TO PARTICIPATE

Not applicable.

## CONSENT FOR PUBLICATION

The authors agree to the publication of all the data involved in this article. No data from other entities are used in this study.

## Data Availability

Not applicable.
